# Speeding disease gene discovery by sequence based candidate prioritization

**DOI:** 10.1186/1471-2105-6-55

**Published:** 2005-03-14

**Authors:** Euan A Adie, Richard R Adams, Kathryn L Evans, David J Porteous, Ben S Pickard

**Affiliations:** 1 Medical Genetics Section, Department of Medical Sciences, The University of Edinburgh, Edinburgh, UK

## Abstract

**Background:**

Regions of interest identified through genetic linkage studies regularly exceed 30 centimorgans in size and can contain hundreds of genes. Traditionally this number is reduced by matching functional annotation to knowledge of the disease or phenotype in question. However, here we show that disease genes share patterns of sequence-based features that can provide a good basis for automatic prioritization of candidates by machine learning.

**Results:**

We examined a variety of sequence-based features and found that for many of them there are significant differences between the sets of genes known to be involved in human hereditary disease and those not known to be involved in disease. We have created an automatic classifier called PROSPECTR based on those features using the alternating decision tree algorithm which ranks genes in the order of likelihood of involvement in disease. On average, PROSPECTR enriches lists for disease genes two-fold 77% of the time, five-fold 37% of the time and twenty-fold 11% of the time.

**Conclusion:**

PROSPECTR is a simple and effective way to identify genes involved in Mendelian and oligogenic disorders. It performs markedly better than the single existing sequence-based classifier on novel data. PROSPECTR could save investigators looking at large regions of interest time and effort by prioritizing positional candidate genes for mutation detection and case-control association studies.

## Background

Over the last twenty years the genes underlying more than a thousand classically Mendelian disorders have been successfully identified. By contrast, only a relatively small number of genetic components of complex traits have been characterized [1].

Regions of interest identified through complex-trait linkage studies regularly exceed 30 centimorgans in size and can contain hundreds of genes. The traditional candidate-gene approach to reducing this number of genes to a manageable level involves attempting to match functional annotation to knowledge of the disease or phenotype under investigation. Unfortunately this approach has been characterized by unsubstantiated and unreplicated claims [2].

Problems arise firstly because the link between genotype and phenotype in complex disorders tends to be weak; matching a single gene's functional annotation to a phenotype is unlikely to be successful unless the gene in question is clearly related to some known pathogenesis of the disease. Secondly, functional annotation of the human genome is incomplete and biased towards better studied genes which have higher levels of annotation. Furthermore, assigning functional annotation is a time-consuming process which is unavoidably error-prone [3,4] and, if taken at face value, misannotated genes can mislead or delay researchers [5].

Van Driel et al. developed a web-based system for automating the annotation based candidate-gene approach [6] that collates expression and phenotypic data from nine different databases and returns genes that conform to investigator-defined criteria. Recently several other candidate-gene identification systems that rely on grouping Gene Ontology (GO) terms have been described [7,8], notably POCUS [9], which finds genes across multiple susceptibility loci that share Interpro [10] domains and GO terms. These systems all rely on functional annotation to make correct predictions, but given that such annotation is incomplete and inherently biased towards a particular subset of genes, a more robust option might be to use sequence-based features instead.

It has been suggested that the genes underlying human hereditary disease share certain distinctive, sequence-based features such as larger gene size [11]. By using machine learning algorithms we aimed to discover such common patterns that could be applied to create an automatic classification scheme capable of identifying genes more likely than not to be involved in disease.

Machine learning has moved rapidly from the field of experimental artificial intelligence to that of applied science. Bioinformatics researchers have been quick to adopt machine learning algorithms in a variety of different situations and their use is now widespread [12]. Lopez-Bigas et al. recently presented a relatively successful decision tree created using such techniques [13] which used amino-acid length and a measure of sequence conservation across species of genes as features to predict genes likely to be involved in hereditary disease. Our approach was related but examined a broader set of features and algorithms, producing a significantly more successful classifier that is able to predict genes involved in both Mendelian and more complex traits. We have also created a web interface to allow researchers to easily classify individual genes or whole regions of the genome and made it freely accessible at .

## Results

### Defining features and building the training set

A set of features was chosen based on a comparative study of ~ 18,000 known genes from Ensembl [14] which are not known to be involved in human disease and the 1,084 Ensembl genes also listed in Online Mendelian Inheritance in Man (OMIM) [15]. The feature set (described in Table 1) reflects the structure, content and phylogenetic extent (the extent to which a gene is conserved back through evolution based on homologs in other species) of each gene examined. We included signal peptide and transmembrane domain predictions; though these are strictly speaking functional attributes they can be calculated with a high degree of accuracy directly from sequence.

Table 2 lists the features we found to be different between the Ensembl genes in OMIM and those not in OMIM. Using the Mann-Whitney U test we found highly significant differences between the gene, cDNA and protein sizes of the two sets (P < 0.001). The genes listed in OMIM were significantly larger and encoded larger proteins; this confirms previous findings [11,13] which noted that the genes and proteins involved in human disease tend to be larger than average. Similarly, we found that the genes listed in OMIM were far more likely to have well conserved best reciprocal hit (BRH) homologs with other species and in particular with mice; this also concurs with previous studies [13,16]. The percentage of gene products that are secreted was much higher in the set of genes listed in OMIM than on average (P < 0.0001) and perhaps unsurprisingly given the larger sizes of genes involved in disease and the correlation between gene size and exon number we found a highly significant difference in the number of exons per gene (P < 0.001 using the Mann-Whitney U test). Genes listed in OMIM had a median of 10 exons while genes not known to be involved in disease had a median number of 8. Genes listed in OMIM were more frequently expressed in specific tissues (P < 0.001) and again this confirms previous findings [17] – however, it was decided to exclude tissue specificity from our feature set in order to avoid potential bias (see Methods).

We also found novel differences between the two sets of genes. There was a small difference (P < 0.028) in the number of CpG islands at the 5' end of genes listed in OMIM and those not, with slightly more genes listed in OMIM having 5' CpG islands, which are associated with both housekeeping genes and to a lesser extent tissue specific genes [18]. There was also a significant difference (P < 0.01) in the length of the 3' UTR between genes listed in OMIM (median 488 bp) and those not involved in disease (median 446 bp). There was also a significant disparity (P < 0.01) in the distance to the nearest neighbouring gene – genes listed in OMIM had a median distance of 52 kb to their neighbours while genes not known to be involved in disease had a median distance of 46 kb. To our knowledge these features have not been previously reported.

**Figure 1:**
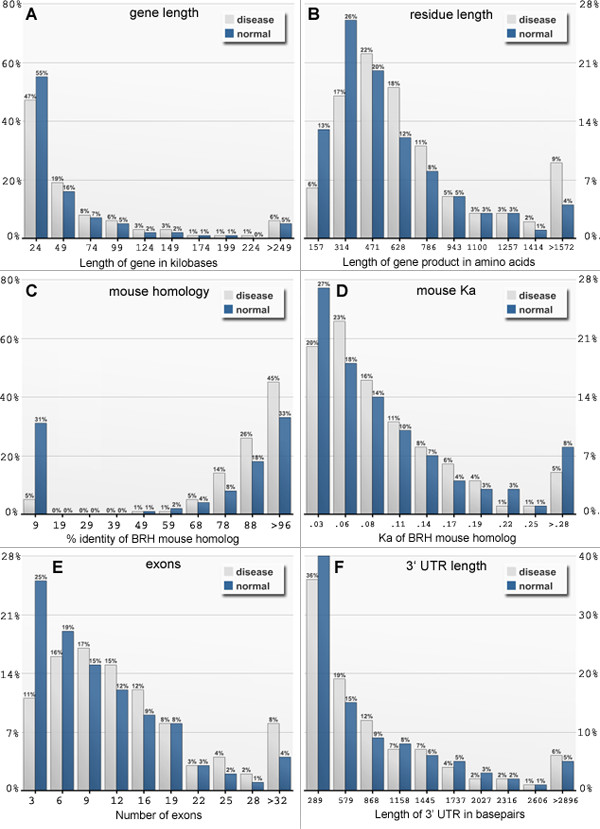
**Histograms of selected features. **Histograms showing distributions of selected features in both "disease genes" (those listed in OMIM) and control genes (those not). Data was binned for graphing purposes. Distributions are shown for (A) gene length in kilobases; (B) protein length in amino acids; (C) % identity of the best reciprocal hit (BRH) homolog in mouse; (D) Ka (a measure of non-synonymous change between species) of the BRH homolog in mouse; (E) number of exons and (F) 3' UTR length in basepairs.

Graphs showing the different distributions of selected features in the two sets are shown in Figure 1. Though some of the differences we found have previously been described in literature, the discrepancy in 3' UTR length has to our knowledge not been examined before and cannot be easily explained in terms of correlation to other, known feature differences. Two other novel features are the distance to the nearest neighbouring gene and the number of exons; both of these are quite strongly correlated to gene size (with Spearman correlation coefficients of 0.69 and 0.71, respectively).

We also studied the number of Interpro domains in each set of genes and found significant differences but concluded that a bias existed towards better studied genes. Therefore we excluded this feature from our study (see Methods).

Automatic classifiers are created by being trained on a set of genes that has already been classified manually. Our training set of genes was made up of the 1,084 genes found in both OMIM and Ensembl (the "disease genes") and 1,084 Ensembl genes not known to be involved in disease (the "control genes") which were selected at random from the larger set of ~ 18,000 as a representative sample.

### Choosing an algorithm

We used Weka [19] as the platform for our machine learning experiments. A variety of different machine learning methods were examined but the alternating decision tree algorithm was chosen as the basis of our classification scheme as it couples high accuracy with a relatively small set of rules [20]. The advantage of decision tree based schemes over other popular algorithms such as k-Nearest Neighbour, Support Vector Machines and Bayesian Networks is that the rules that are produced for classifying instances can be interpreted more easily by non-expert users. This is particularly true for the alternating decision tree algorithm, which typically produces trees that are just as predictive as those created by more traditional decision tree algorithms but that are far more concise and thus easier to understand. Alternating decision trees also allowed us to measure the contribution of each feature to the final classification of a gene, which might provide insight into the essential differences between those genes more and less likely to be involved in disease.

Alternating decision trees are created by adding rules to the tree in an iterative fashion in the order of their predictive power, with the more effective rules being added first. These rules are automatically derived from the differences between the disease and control genes in the training set provided. A new rule is added to the tree either as a new "node" or as a child of an existing node. With Weka, the number of nodes to add to the tree is specified by the user before training begins. Too few nodes and the tree will be sparse, without enough cumulative discriminatory power to make confident classifications. Too many nodes, on the other hand, will result in an overly-complex tree where later nodes with weak predictive power can distort the effects of earlier, more predictive nodes.

On the basis of past experience we chose to limit the size of our alternating decision tree to fifteen nodes, which is a good balance of predictive power and complexity. As each node represents a rule that tests a single sequence feature, this meant that of the two dozen sequence features available a maximum of fifteen would be used in the final tree.

**Figure 2:**
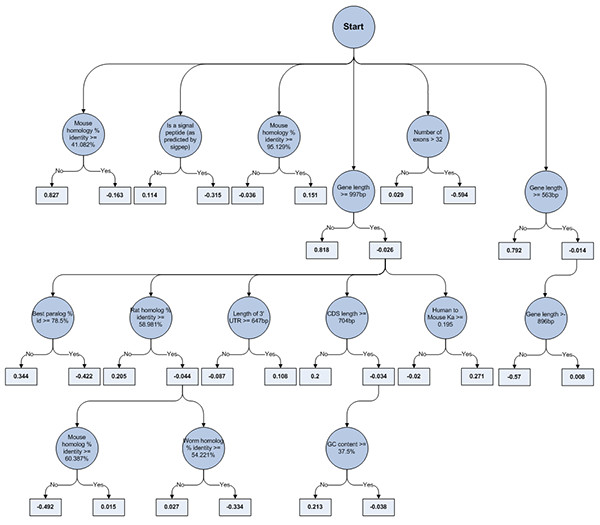
**The alternating decision tree. **The alternating decision tree used to classify instances. A gene is classified with the tree by beginning at the node marked "Start" and then following each branch in turn. Upon reaching a node which contains an assumption the "yes" or "no" branch is followed as appropriate. If the relevant feature is "unknown", neither branch is followed. Adding up each of the numbers in rectangles that are encountered along the way results in a final score which reflects the relative confidence of the classification. The classification itself is based on the sign of the score.

An alternating decision tree with fifteen nodes was produced by training on the training set of genes and is shown in Figure 2. We also produced trees with ten and twenty nodes for comparison and discovered using the measurements described below that classifier performance was indeed poorer than with the fifteen node version (details not shown).

### The alternating decision tree

A gene is classified with the tree in Figure 2 by beginning at the node marked "Start" and then following each branch in turn. Upon reaching a node that contains an assumption – for example, that the gene length is larger than a given number – the "yes" or "no" branch is followed as appropriate. If the relevant feature – the paralog percentage identity, for example – is "unknown", neither branch is followed. Adding up each of the numbers in rectangles that are encountered along the way results in a final score that reflects the relative confidence of the classification. The classification itself is based on the sign of the score – if negative the gene is generally more likely to be involved in hereditary disease, if positive the gene is generally less likely to be involved in hereditary disease.

We tested the classifier on our training set of genes. 77% of the disease genes were correctly identified (that is to say had a negative score). In contrast, 42% of the 1,084 control genes were classified as disease genes (were false positives). As this is a predictive approach – we cannot say a priori how much of the genome and thus the representative sample in the training set is made up of genes that are involved in disease but are not yet characterized – at least some of these apparently incorrect classifications are likely to be correct.

We ran a tenfold cross-validation test to get a conservative estimate of how our classifier might perform on unseen data. Tenfold cross-validation is a widely used technique in machine learning and involves partitioning the whole training set into ten independent "folds" each with the same balance of disease genes and control genes. The classifier is trained on nine of the partitions and tested on the remaining partition. This is repeated until each partition has been tested on a new classifier built with the remainder of the training set and simulates the performance of the chosen algorithm and feature set on unseen data. On average, 70% of the disease genes were correctly identified during cross-validation with 43% of control genes classified as false positives. This is comparable to the results obtained by Lopez-Bigas et al. [13] during cross-validation. Table 3 contains more detailed statistics relating to classifier performance.

**Figure 3:**
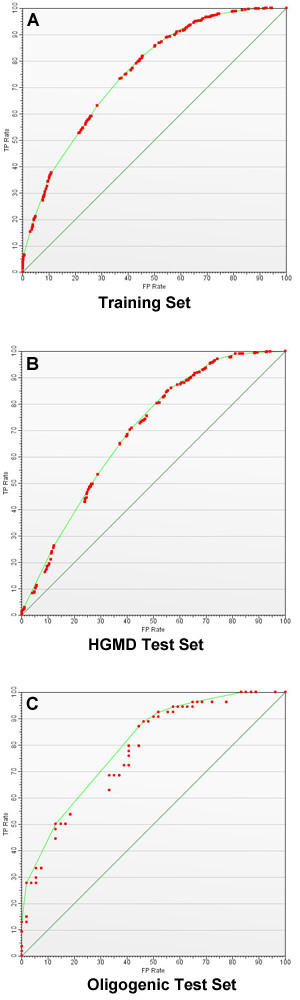
**Receiver Operating Characteristic (ROC) curves. **Receiver Operating Characteristic (ROC) curves for the training set (A) and the two test sets (B and C). The true positive rate is measured along the y-axis and the false positive along the x-axis. The area under the resulting curve is a measure of classifier performance.

As the alternating decision tree outputs a score that can be thresholded, it is a relatively simple matter to increase specificity (precision) at the expense of sensitivity (recall). Receiver Operating Characteristic (ROC) curves can be used to visualise classifier performance with different combinations of specificity and sensitivity. The x-axis of a ROC curve represents the fraction of false positives and the y-axis the fraction of true positives in the classifier results. As the number of true positives (sensitivity) increases, so too does the number of false positives (decreasing specificity). Figure 3 shows the ROC curves for the classifier on the training set and the two test sets which are described below.

Table 4 shows the relative importance of the eleven different sequence features used by the classifier. We calculated these values by testing the classifier on our training set and, for each gene, keeping track of the percentage contribution of each feature to the final score. These percentages were then averaged out over all genes predicted as likely to be involved in disease. It should be noted that while the percentages given accurately reflect the relative contribution of each feature *to our classifier *they are meaningless when taken out of context; by themselves, for example, GC content and the % identity of a worm homolog are not necessarily equally predictive features for distinguishing between genes that are more likely and those less likely to be involved in disease.

We implemented our classifier as a standalone script in Perl and designed an associated web interface to aid in the interpretation of the results produced. The resulting software is named PROSPECTR (for **PR**i**O**rization by **S**equence &**P**hylog**E**netic features of **C**andida**T**e **R**egions) and is freely accessible together with training and test sets of genes at . The web interface allows researchers to quickly obtain scores for regions of the genome or individual genes of interest.

### Further testing

Evaluating classifier performance on the training set alone is potentially misleading as over-fitting may have occurred. Over-fitting happens when a classifier generalises only to the extent necessary to work well on the training data, resulting in poor performance on data that was not seen during the training process. Cross-validation provides a measure of the performance of our approach in general, but doesn't reflect actual PROSPECTR performance accurately as the alternating decision trees created for each fold are different. We therefore created two test sets independent of the training set.

The first independent test set (the "HGMD set") contained 675 genes associated with disease listed in the Human Gene Mutation Database [21] and 675 genes not known to be involved in disease that were picked at random from Ensembl. The second (the "oligogenic set") contained 54 genes not known to be involved in disease and picked at random from Ensembl and 54 genes not listed in OMIM but associated with different oligogenic disorders including inflammatory bowel disease, Parkinsons, Retinitis Pigmentosa and autosomal recessive limb-girdle muscular dystrophy.

We were unable to obtain a sizeable, reliable set of genes involved in complex traits; this meant that classifier performance could not be tested on the components of complex disease. This may change in the future as resources such as the Genetic Association Database [22] develop further and more association data becomes accessible.

71% (478) of the disease genes from the HGMD set and 72% (39) of the genes from the oligogenic set were correctly identified by the classifier, with 42% (282) and 41% (22) of control genes misclassified respectively. These results are similar to those obtained on the training set, suggesting that over-fitting did not occur. They also suggest that our sequence-based approach works equally well for finding genes involved in both oligogenic and monogenic disorders.

Lopez-Bigas et al [13] used a larger set of disease genes during training. Only 260 of the genes from the HGMD set were independent of the training sets of both PROSPECTR and the Lopez-Bigas classifier. As a comparative measure, these 260 genes were scored using both classifiers. PROSPECTR correctly identified 72% (189) of the disease genes while the Lopez-Bigas classifier identified 47% (123).

PROSPECTR, however, had a higher false positive rate, categorising ~ 44% of the whole human genome as likely to be involved in disease while the Lopez-Bigas classifier categorised ~ 31% of the whole human genome as likely to be involved in disease. To see how this might have affected recall we calculated the Kappa statistic [23] for the results from both classifiers. The Kappa statistic is a measurement of agreement between predicted and actual classifications and takes false positive rates into account. It is a number between 1 (symbolising perfect agreement between predicted and actual classifications) and 0 (symbolising no agreement). On the independent HGMD set of 260 genes and assuming a false positive rate of 31%, the Lopez-Bigas classifier had a Kappa statistic of 0.158 while PROSPECTR assuming a false positive rate of 44% had a Kappa statistic of 0.282, a factor of almost twofold. This suggests that PROSPECTR is substantially more adept than the Lopez-Bigas classifier at correctly classifying unseen data.

By ranking genes by score in descending order, it is possible for PROSPECTR to create a list of genes for any given locus the top of which is enriched for genes that have a higher probability of being involved in disease.

To test this we took the HGMD set and for each gene created an artificial locus 30 Mb in size consisting of the gene from the HGMD set and all known genes within 15 Mb on either side on the same chromosome. The gene taken from the HGMD set was in each case designated the "target gene" and by scoring each gene in the artificial locus and then ranking them we were able to see where the target gene appeared in the ordered list that was created.

For the 675 genes from the HGMD set the average number of genes per list was 202. Target genes were in the top 5% of the ordered list 68 times (10% of the time), top 10% 125 times (18%), top 50% 510 times (75%) and the top 75% 639 times (94%).

We repeated the procedure for the 1,084 genes listed in OMIM from our training set. The average number of genes per list was 198 and target genes were in the top 5% 171 times out of 1084 (15%) and the top 50% 873 times (80%).

The genes from the training and HGMD sets are mostly Mendelian monogenic disorders; to see if the classifier was equally successful at enriching loci involved in more complex diseases we took the list of 219 genes likely to be involved in oligogenic disorders used as a test set by POCUS [9].

**Figure 4:**
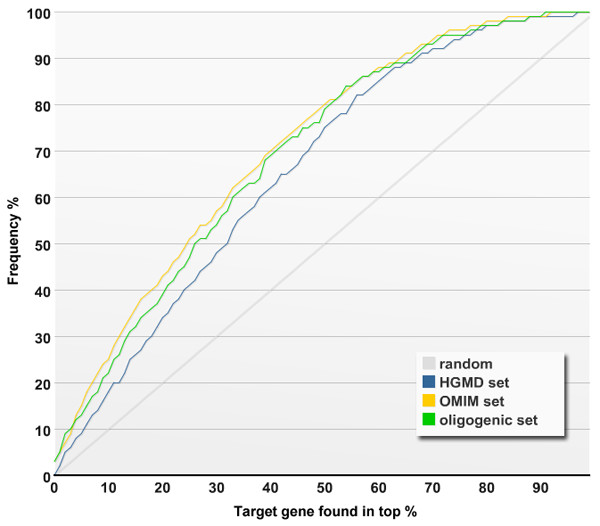
**Performance over artificial loci. **Relative performance on the sets of artificial loci created from the training set (yellow line), HGMD test set (the blue line) and oligogenic test set (the green line). The gray line represents the value expected if there had been no enrichment. The x axis represents the % of the ranked list in which the target gene was found; the y axis represents how frequent that occurrence was. For example, in the training set (the yellow line) the target gene was in the top 30% of the ranked list around 56% of the time.

For these 219 genes involved in oligogenic disorders the average number of genes per list was 209 and the target gene was in the top 5% 29 times (13.4%) and the top 50% 172 times (79%). Figure 4 shows a graphical representation of these results.

### Performance on different types of mutation

Gene records from the HGMD contain information about the number of different mutations associated with any phenotypes linked to that gene, split into three types: nucleotide substitutions, micro-lesions and gross lesions (including repeat variations and complex rearrangements). For example, the Huntington gene (*HD*) is recorded as being implicated in Huntington disease, which is associated with a gross lesion. The Haemoglobin beta gene (*HBB*) is recorded as being implicated in sickle cell anaemia, associated with nucleotide substitutions.

Of the HGMD set we used to test performance, 297 genes were associated with nucleotide substitutions only, 55 with gross lesions only and 27 with micro-lesions only. We tested each subset separately to determine if the underlying cause of disease influenced PROSPECTR's performance.

We found that 75% and 77% of the genes involved in disease and associated only with nucleotide substitutions or only with micro-lesions, respectively, were correctly identified by PROSPECTR. However, only 54% of the genes involved in disease and associated only with gross lesions were identified. This suggests that the decision tree used by PROSPECTR is better at identifying genes likely to be involved in disease because of small or point mutations than genes involved in disease because of more drastic events like gross deletions, insertions and chromosomal aberrations.

### Whole genome analysis

PROSPECTR was used to score every known gene in the Ensembl database on the likelihood that it is involved in human hereditary disease. We normalised the score *α *given to each gene with the equation



where gamma (*γ*) represents Euler's constant so that it fell between 0 and 1 with higher scores suggesting a higher likelihood of involvement in disease.

97 genes had a score over 0.75, of which 36 (~ 33%) are listed in either the HGMD or OMIM and are thus already known to be involved in disease (this represents a more than threefold enrichment; Ensembl contained ~ 19,500 known genes of which ~ 9% were known disease genes). By contrast, in the set of 4,357 genes which scored less than 0.3 only ~ 0.8% (35 genes) are already known to be involved in disease.

A list of the 61 genes that scored higher than 0.75 but are not already known to be involved in disease is included as supplementary material (see Additional File 1). By searching for references in PubMed we discovered that 9 of these genes (~ 15% of the total) are already candidates for involvement in diseases including Alzheimers (*ABCA2*), osteoporosis (*COL4A1 *and *COL4A2*) and schizophrenia (*SLIT3*).

## Discussion

### Relative performance

PROSPECTR has a number of advantages over existing classification schemes designed to differentiate between genes more and less likely to be involved in disease.

PROSPECTR appears to perform significantly better on unseen data than the decision tree classifier presented by Lopez-Bigas et al. The Lopez-Bigas classifier is less likely to be useful as a predictive tool for two, related reasons: firstly, it achieves perfect accuracy when tested on the training set, even though the training set is known to be inconsistent. The Lopez-Bigas classifier suggests that ~ 31% of the genome is made up of predicted disease genes. Thus if genes were picked at random to make up the control set during training it should be assumed that ~ 31% of them are actually disease genes which have not yet been characterized. Perfect accuracy on the training set is therefore undesirable – by ignoring the possibility that the set of control genes might contain disease genes the classifier loses flexibility and predictive power. Secondly, the fact that all uncharacterized disease genes were predicted as being control despite their presumed strong similarity (in terms of sequence features) to other disease genes suggests that it is highly likely that at least some degree of overfitting occurred, which would further impair performance on novel data.

PROSPECTR's use of a spread of sequence-based features representing the structure, content and phylogenetic extent of candidate genes allows investigators to see exactly which features are contributing the most towards a particular classification. In addition, it requires no detailed phenotypic knowledge of the disease in question and can score whole chromosomes in minutes. The use of sequence-based features avoids the bias inherent in current functional annotation, where better studied genes are far more likely to have better and more extensive annotation. Furthermore, by relying less on phylogenetic conservation we reduce the amount of potential bias from imperfect homology prediction (see Eliminating Bias in Methods).

### Classifier mechanics

Other researchers have examined some of the sequence-based differences between genes listed in OMIM and genes not known to be involved in disease but there have been no comprehensive studies. The data we present here collates all of the known sequence-based differences and introduces some new ones – for example, the differences in 3' UTR length between the two sets of genes are statistically significant and, though a correlation with gene size exists, it is relatively weak (a Spearman correlation coefficient of 0.35). Further research is needed to suggest how all of these differences might relate to, for example, a gene's relative importance or position in a protein-protein interaction map or biological pathway.

The length of the 3' UTR is thought to be related to translational efficiency and mRNA stability [24], which in turn affects the level of expression of the gene. Two other novel sequence-based features where we found significant differences between disease and non-disease genes – the distance to the nearest neighbouring gene and the number of exons – might also be directly related to expression levels [25]. In this work we were able to confirm the suggestions from previous studies that there is a significant difference in tissue specificity between disease and non-disease genes [17] – perhaps similar differences exist between the two sets in patterns of overall gene expression levels.

It seems remarkable that disease genes would share sequence features to such an extent. In particular gene length and protein length when taken together as features for an alternating decision tree classifier with fifteen nodes can be reasonably predictive (69% of disease genes correctly classified from the training set with 51% misclassification, details not shown).

A complex web of correlations exists between gene and protein size, levels of expression and rates of evolution, which perhaps explains why predictive power remains relatively high after removing features other than gene and protein size. Additionally, larger genes might simply be bigger targets for mutation [13], or be more likely to have sequence features like overlapping gene groups, multiple amino acid runs [26] and motifs associated with mutational hotspots which might increase the chance of them succumbing to some disease causing mutation.

An alternative hypothesis is that PROSPECTR does not predict genes likely to be involved in disease at all, but the opposite: it derives its predictive power from discounting those genes which are unlikely to be involved in disease as mutations usually result in a phenotype which is either lethal (in which case we wouldn't class it as a disease gene), undetectable (in which case we couldn't class it as a disease gene) or very weak (in which case the classification of the gene would be debateable).

We have shown that PROSPECTR performs well on an oligogenic test set. However, one might expect the biological mechanisms of cause and effect to differ between simple Mendelian and more complex traits and therefore the classifiers dealing with either type may also have to differ. Currently no sizeable dataset of genes involved in complex disease exists; until one is created and examined we cannot tell how PROSPECTR will perform when used to find the genes underlying complex disease. We would thus advise caution when using PROSPECTR to search for genes involved in complex traits.

### Future directions

PROSPECTR can create lists of genes the tops of which are enriched for those genes that are likely to be involved in human disease. Substantial enrichment is highly likely with this sequence-based approach, although investigators still need to carry out functional comparisons and fine scale mapping to reduce lists to one or two candidates for each region of interest. By contrast, functional classifiers might present only a handful of high quality suggestions for each of the regions studied but equally might not return the target gene at all as their threshold for successful detection is too high.

One way of speeding the candidate gene discovery process further without sacrificing accuracy might be to combine existing techniques that use functional annotation with a sequence-based approach similar to the one we describe here. It may be possible to create a combined classifier greater than the sum of its parts by lowering the threshold of a successful functional annotation based classifier and then dismissing false positives using a sequence-based approach.

The alternating decision tree used in PROSPECTR was trained using all genes from OMIM and as such is suited for general use. However, there might well be some value in creating custom classifiers targeted to a particular area of interest; for example, genes involved in neurological disorders. If the training set was still sufficiently large enough to be representative then one might expect more precision when scoring candidate genes in similar disorders. As a first step towards this we have made instructions for creating custom classifiers available on the PROSPECTR website.

## Conclusion

On average, PROSPECTR successfully enriches lists of candidate genes 2-fold ~ 77% of the time, 5-fold ~ 37% of the time and 25-fold ~ 11% of the time. It does so for both monogenic and oligogenic disorders and on the basis of a compact set of rules which look at sequence-based features. These features reflect the structure and content of the genes in question as well as the phylogenetic extent and are much less likely to be biased towards better studied genes than manual annotation.

The rules involved are easily interpretable which gives some insight into how the classifier works and the importance of various features relative to each other, signposting new avenues of investigation into the differences between the types of disease and non-disease genes.

We predict that the growing availability of relevant protein-protein interaction data and better functional annotation will greatly improve candidate identification techniques for oligogenic and complex disorders, as shared or compensated pathways become clearer. However, robust genome-wide functional annotation is still some way off. In the meantime, using PROSPECTR as a quick, unbiased method to rank genes in order of their likelihood of involvement in disease could save investigators much time and effort when examining larger regions of interest, prioritizing candidates for more in-depth functional characterization, mutation detection and case control studies.

Our implementation of PROSPECTR is readily available on the web at .

## Methods

We used Online Mendelian Inheritance in Man (OMIM) and the Human Gene Mutation Database (HGMD) to obtain lists of disease genes and MySQL client access to Ensembl to retrieve the sequences for those genes. We also used Ensembl to provide genes as a control set by selecting reasonably sized representative sets at random. The genes in the control set were not listed in either OMIM or the HGMD.

To create our initial feature set we collated information from Ensembl, NCBI's Homologene, Interpro [10], SwissPROT and the Novartis Gene Expression Atlas [27]. This initial feature set included all of the features listed in Table 1 as well as information relating to tissue expression and protein domain distribution. The relevant information for all known genes in Ensembl was stored in a local MySQL database. We then compared features from a set of 1,084 genes listed in OMIM with a representative sample from the control set made up of ~ 18,000 genes from Ensembl not listed in OMIM. Features that were considered to have a reasonable degree of predictive power were selected to create the feature set to be made available to the alternating decision tree algorithm. To calculate the tissue specificity of each gene we used the same method as Winter et. al [17].

We used Weka as the platform for building our classifier. Weka is free, open source Java application and is readily available on the internet [19]. Experiments were carried out using the Explorer interface to Weka using the ADTree classifier. The accuracy, precision, recall, AUC (the area under the ROC curve; used as a performance metric) and Kappa statistics in Table 3 were obtained directly from Weka. We used custom Perl scripts to create artificial loci and then rank the scores of the genes they contained. We wrote the PROSPECTR software using Perl and the Apache web server.

### Eliminating bias and sources of error

We studied the feature set for potential bias. In particular, the number of Interpro domains described on each gene appeared to have more to do with a bias towards better studied genes than with disease gene association. When we compared the number of Interpro domains between the OMIM genes and a group of genes not known to be involved in disease but with at least one reference in literature (according to their SwissPROT record) no significant differences were found. We therefore eliminated any features based on Interpro domains as potentially biased.

Though highly significant differences in tissue expression patterns were detected, it was decided to exclude the tissue specificity feature from the training set as it introduced a bias towards disease genes; reliable, normalised tissue expression data was available for ~ 95% of genes implicated in disease but only two thirds of control genes. Genes without the relevant data could have been ignored or had a best guess value assigned to them, but this would have undermined the classifier's usefulness for detecting novel disease gene candidates and introduced new sources of bias.

Determining phylogenetic extent by looking at homologs is also a potential source of bias as disease genes are better characterized and more transcript evidence is available. Imperfection in gene prediction is a major hindrance to accurate orthology prediction [16]. On average, around a third (~ 34%) of the predictive power of our classifier comes from features related to phylogenetic extent.

There exists a possibility that the set of OMIM genes that make up the training set is itself biased towards genes containing features which somehow make linking a disease to an allele of that gene easier. However, we believe this to be unlikely.

Firstly, it is important to remember that, at least with Mendelian disorders, it has been the goal of identifying the gene behind a particular common disease that has driven research, not matching diseases to genes that are easier to find, clone or characterize. Secondly, although the OMIM database has been collecting information about Mendelian disorders for many years the majority of confirmed disease genes have been added more recently after having been mapped and characterized with the help of publicly available sequence data and modern molecular biology techniques – none of which are obviously biased towards particular sequence features. Finally, given the combined size (~ 1,700 genes) of the training and test datasets it seems reasonable to assume that we are working with a representative sample of disease genes.

## Authors' contributions

BP, RA and EA conceived of the study, which was coordinated by BP. EA carried out the work with Perl and Weka with help from RA who also participated in testing. KE and DP participated in the analysis of the results and stimulated discussion. All authors helped to draft the manuscript.

## Supplementary Material

Additional File 1 The 61 top scoring genes not known to be implicated in disease. Click here for file

## Figures and Tables

**Table 1:** The feature set. The list of features which were made available to the machine learning application (Weka) to build the alternating decision tree.

**Feature**	**Source**	**Description**
Gene length	EnsemblMart 22.1	Length of gene in bp.
CDS length	EnsemblMart 22.1	Length of coding sequence in bp.
cDNA length	EnsemblMart 22.1	Length of complementary DNA in bp.
Protein length	EnsemblMart 22.1	Length of protein in aa.
Length of 3' UTR	EnsemblMart 22.1	The length of the 3' untranslated region (UTR) in bp
Length of 5' UTR	EnsemblMart 22.1	The length of the 5' untranslated region (UTR) in bp
Distance to nearest neighbouring gene	EnsemblMart 22.1	Distance to the next known gene on the same chromosome on either strand in bp.
Number of exons	EnsemblMart 22.1	Number of exons in the gene.
GC	EnsemblMart 22.1	GC content (as a %) of gene
Transmembrane	EnsemblMart 22.1	Prediction of transmembrane domains (1 for yes or 0 for no)
Signal peptide	EnsemblMart 22.1	Prediction of signal peptide (1 for yes or 0 for no)
Paralog	EnsemblMart 22.1	If the gene has a paralog in the human genome (1 for yes or 0 for no)
Paralog % identity	EnsemblMart 22.1	% protein identity of best paralog in the human genome. Genes without paralogs have "unknown" entered here.
Mouse homolog % identity	Homologene	% protein identity of mouse homolog. Genes without a mouse homolog have "0" entered here.
Rat homolog % identity	Homologene	% protein identity of rat homolog. Genes without a rat homolog have "0" entered here.
Worm homolog % identity	Homologene	% protein identity of worm homolog (potentially 0, see above)
Fly homolog % identity	Homologene	% protein identity of fly homolog (potentially 0, see above)
Yeast homolog % identity	Homologene	% protein identity of yeast homolog (potentially 0, see above)
Arabidopsis homolog % identity	Homologene	% protein identity of Arabidopsis homolog (potentially 0, see above)
Mouse homolog Ka	Homologene	Measure of non-synonymous changes between human and mouse homolog.
Mouse homolog Ks	Homologene	Measure of synonymous changes between human and mouse homolog.
Mouse homolog Ka / Ks	Homologene	Ratio of above two fields.
CpG island at 3' end of gene	EnsemblMart 22.1	If a CpG island exists at the 3' end of the gene (1 or 0)
CpG island at 5' end of gene	EnsemblMart 22.1	If a CpG island exists at the 5' end of the gene (1 or 0)

**Table 2:** Significant differences between the control set and disease set of genes. The features found to be significantly different between Ensembl genes found in OMIM and those not in OMIM. Significance was calculated using the Mann-Whitney U test unless otherwise noted.

**Feature**	**Median in control set**	**Median in disease set**	**Significance**
Gene length	19 k	27 k	P < 0.001
cDNA length	2,126 bp	2,442 bp	P < 0.001
Protein length	383 aa	494 aa	P < 0.001
3' UTR length	446 bp	488 bp	P < 0.01
Exon number	8	10	P < 0.001
Distance to neighbouring gene	46 kb	52 kb	P < 0.01
Protein identity with BRH in mouse	80%	87%	P < 0.001
Gene encodes signal peptide	17%	35%	P < 0.0001 (calculated using the chi squared test)
5' CpG islands	12%	16%	P < 0.028 (calculated using the chi squared test)

**Table 3:** More detailed classifier performance statistics. For each set of genes tested, five statistics that reflected performance were calculated. Accuracy is the overall accuracy of the classifier; precision reflects the classifier's specificity and recall reflects classifier sensitivity. The area under curve (AUC) is the area underneath the ROC curve drawn for each set of genes (see Figure 3) and represents classifier performance across all combinations of sensitivity and specificity. It ranges from 0 to 1, where 1 represents 100% accuracy, 0.5 represents performance no better than random and 0 represents 0% accuracy. The Kappa statistic is a measurement of agreement between predicted and actual classifications and takes false positive rates into account. It is a number between 1 (symbolising perfect agreement between predicted and actual classifications) and 0 (symbolising no agreement).

**Test Set**	**Nodes in tree**	**Accuracy**	**Precision**	**Recall**	**AUC**	**Kappa**
Training (OMIM) set	15	67%	65%	77%	0.75	0.35
10 × cross validation	15	63%	62%	70%	0.70	0.27
HGMD set	15	64.5%	63%	71%	0.69	0.29
Oligogenic set	15	65%	63%	72%	0.76	0.31

**Table 4:** Relative contribution of each feature to classification as disease gene. An estimate of the relative contribution of each sequence feature in the final score used by the alternating decision tree for classifying genes as being involved in disease. The percentages are based on the average absolute contribution to the cumulative absolute score of each disease gene in the training set.

**Feature**	**% Contribution to final score**
Signal peptide	23%
Mouse homolog % identity	21%
Length of 3' UTR	12%
Number of exons	7%
Rat homolog % identity	7%
Worm homolog % identity	6%
GC	6%
CDS length	5%
Gene length	4%
Mouse homolog Ka	3%
Paralog % identity	2%

## References

[B1] Glazier AM, Nadeau JH, Aitman TJ (2002). Finding Genes That Underlie Complex Traits. Science.

[B2] McCarthy M, Smedley D, Hide W (2003). New methods for finding disease-susceptibility genes: impact and potential. Genome Biology.

[B3] Devos D, Valencia A (2001). Intrinsic errors in genome annotation. Trends in Genetics.

[B4] Gilks WR, Audit B, De Angelis D, Tsoka S, Ouzounis CA (2002). Modeling the percolation of annotation errors in a database of protein sequences. Bioinformatics.

[B5] Pallen M, Wren B, Parkhill J (1999). 'Going wrong with confidence': misleading sequence analyses of CiaB and ClpX. Molecular Microbiology.

[B6] Van Driel MA, Brunner HG, Leunissen JAM, Kemmeren PPCW, Cuelenaere K (2003). A new web-based data mining tool for the identification of candidate genes for human genetic disorders. European Journal of Human Genetics.

[B7] Freudenberg J, Propping P (2002). A similarity-based method for genome-wide prediction of disease-relevant human genes. Bioinformatics.

[B8] Perez-Iratxeta C, Bork P, Andrade MA (2002). Association of genes to genetically inherited diseases using data mining. Nature Genetics.

[B9] Turner FS, Clutterbuck DR, Semple CAM (2003). POCUS: mining genomic sequence annotation to predict disease genes. Genome Biology.

[B10] Mulder NJ, Apweiler R, Attwood TK, Bairoch A, Barrell D, Bateman A, Binns D, Biswas M, Bradley P, Bork P (2003). The InterPro Database, 2003 brings increased coverage and new features. Nucl Acids Res.

[B11] Smith NGC, Eyre-Walker A (2003). Human disease genes: patterns and predictions. Gene.

[B12] Kapetanovic IM, Rosenfeld S, Izmirilan G (2004). Overview of Commonly Used Bioinformatics Methods and Their Applications. Ann NY Acad Sci.

[B13] Lopez-Bigas N, Ouzounis CA (2004). Genome-wide identification of genes likely to be involved in human genetic disease. Nucl Acids Res.

[B14] Hammond MP, Birney E (2004). Genome information resources – developments at Ensembl. Trends in Genetics.

[B15] Hamosh A, Scott AF, Amberger J, Bocchini C, Valle D, McKusick VA (2002). Online Mendelian Inheritance in Man (OMIM), a knowledgebase of human genes and genetic disorders. Nucl Acids Res.

[B16] Huang H, Winter E, Wang H, Weinstock K, Xing H, Goodstadt L, Stenson P, Cooper D, Smith D, Alba MM (2004). Evolutionary conservation and selection of human disease gene orthologs in the rat and mouse genomes. Genome Biology.

[B17] Winter EE, Goodstadt L, Ponting CP (2004). Elevated Rates of Protein Secretion, Evolution, and Disease Among Tissue-Specific Genes. Genome Res.

[B18] Gardiner-Garden M, Frommer M (1987). CpG islands in vertebrate genomes. Journal of Molecular Biology.

[B19] Frank E, Hall M, Trigg L, Holmes G, Witten IH (2004). Data mining in bioinformatics using Weka. Bioinformatics.

[B20] Freund Y, Mason L The Alternating Decision Tree Learning Algorithm. Proceedings of the Sixteenth International Conference on Machine Learning.

[B21] Stenson PD, Ball EV, Mort M, Philips AD, Shiel JA, Thomas NST, Abeysinghe S, Krawczak M, Cooper DN (2004). Human Gene Mutation Database (HGMD^®^): 2003 update. Human Mutation.

[B22] Becker KG, Barnes KC, Bright TJ, Wang SA (2004). The Genetic Association Database. Nature Genetics.

[B23] Forbes AD (1995). Classification algorithm evaluation: five performance measures based on confusion matrices. Journal of Clinical Monitoring.

[B24] Tanguay RL, Gallie DR (1996). Translational efficiency is regulated by the length of the 3' untranslated region. Molecular Cellular Biology.

[B25] Chiaromonte F, Miller W, Eric E (2003). Gene Length and Proximity to Neighbors Affect Genome-Wide Expression Levels. Genome Res.

[B26] Karlin S, Chen C, Gentles AJ, Cleary M (2002). Associations between human disease genes and overlapping gene groups and multiple amino acid runs. PNAS.

[B27] Su AI, Cooke MP, Ching KA, Hakak Y, Walker JR, Wiltshire T, Orth AP, Vega RG, Sapinoso LM, Moqrich A (2002). Large-scale analysis of the human and mouse transcriptomes. PNAS.

